# Prevalence of Overweight and Obesity among Libyan Men and Women

**DOI:** 10.1155/2019/8531360

**Published:** 2019-07-15

**Authors:** H. Lemamsha, G. Randhawa, C. Papadopoulos

**Affiliations:** ^1^Faculty of Medical Sciences, University of Omar Al-Mukhtar, Al-Bayda Campus, Labraq Road, Al-Bayda B1L12, Libya; ^2^Institute for Health Research, University of Bedfordshire, Putteridge Bury Campus, Hitchin Road, Luton LU2 8LE, UK

## Abstract

Libya is following the trend observed in developing countries of steadily becoming more obese, such that obesity in Libya has reached epidemic proportions in the twenty-first century. The prevalence of obesity in Libya has more than doubled in the last three decades, with the numbers of overweight and obese adults being continuing to grow. Therefore, this study aimed to estimate and describe the prevalence of overweight and obesity among Libyan men and women. A cross-sectional survey was conducted to examine the prevalence of overweight and obesity among the Libyan population. A multistage sampling technique was employed to select 401 Libyan adults randomly from the Benghazi electoral register. Qualified nurses were allocated to take anthropometric measurements (including visceral fat and Body Mass Index (BMI)) from participants using the Segmental Body Composition Analyser and a portable Stadiometer. The response rate achieved in this cross-sectional study was 78%. Four hundred and one Libyan adult, aged 20-65 years, participated; 253 were female (63%). The prevalence of obesity, overweight, and normal weight among Libyan adults was 42.4%, 32.9%, and 24.7%, respectively. The results also revealed that approximately 75.3% of Libyan adults were overweight and obese, and the prevalence of overweight and obesity in women was significantly higher than that in men (the prevalence of overweight was 33.2% in women compared to 32.4% in men, while the prevalence of obesity was 47.4% in women compared to 33.8% in men, respectively). The findings of this study confirmed that obesity and overweight are the fastest growing issues and have become one of the most serious public health challenges confronting the Libyan authorities. As the obesity epidemic in Libya continues to escalate, with a complete absence of prevention interventions to reduce obesity, more research is desperately needed to follow the trend of gender difference in the prevalence of overweight and obesity among Libyans adults across the Libyan state to improve the effective interventions for preventing obesity.

## 1. Background

Obesity is a leading cause of various noncommunicable diseases (NCDs), including life-threatening and nonfatal diseases; obesity also leads to premature death and disability in adulthood [1–3]. According to the WHO (2018) [3], the worldwide obesity prevalence has increased to almost tripled since the year 1975; as a result, approximately more than 1.9 billion adults worldwide were overweight, with over 650 million being clinically obese [3]. Given that obesity is a growing public health concern worldwide [4, 5] that has serious implications on both individuals and societies [6, 7], the World Obesity Day was emerged and launched in Latin America in 1998 [8].

A large body of literature has shown that there is a paucity of studies about the prevalence of obesity in adults in Arab countries in the North African region, particularly Libya [9, 10]. Since the discovery of oil in 1959, Libya has witnessed dramatic social, economic, and political transformations in the past five decades, including notable shifts in dietary and physical activity patterns [11]. It is likely that obesity has profound consequences for Libyan society as a whole [12]. Consequently, it is important to understand more about the trends in the prevalence of obesity among Libyan adults because of the detrimental effects of obesity on the individual's physical and mental health and on social and family life, life expectancy, and the financial burden it places on the state.

Obesity has become a serious health concern within the Libyan context because obesity poses a major risk for serious diet-related deadly diseases leading to disability and premature death which might attribute to the rates of obesity dramatically increasing in the Libyan adult population [13, 14]. Furthermore, obesity causes various psychological, social, and economic implications [13, 15], which lead to increased morbidity, mortality, and economic losses. Therefore, it is convincible to conduct survey research to estimate the accurate assessment of overweight and obesity of prevalence among adults in the Libyan population. According to Libyan national health survey and WHO NCDs are the leading cause of death in Libya; they estimated more than 76% of deaths annually, which is the highest percentage in the Middle East and North Africa (MENA) region [16, 17].

Apart from the psychological and sociological consequences of obesity, obesity places a financial burden on the Libyan state as a whole. The cost of obesity in Libya in 2015 was projected to be 5.5 billion Libyan Dinar (LYD) (direct costs only) (3.09 billion British pounds) which is roughly 65% of the countries health budget [18–20]. The direct costs are spent mainly on the treatment of obesity-related comorbidities including the travelling to seek treatment in European countries because many Libyans perceive Libyan health services to be unsatisfactory, while indirect costs include work-days and productivity lost, unemployment [20, 21], benefit payments, premature retirement, and premature mortality, the costs of which have not yet been estimated [16, 20, 21]. With the rise in obesity, these figures are likely to increase.

Libya is following the trend observed in developing countries of steadily becoming more obese, such that obesity in Libya has reached epidemic proportions in the twenty-first century; within all ages obesity is continuing to grow [11, 16, 22–24]. The obesity rate (BMI ≥30 kg/m^2^) has more than doubled in the last three decades, from 12.6% in 1984 to 30.5% in 2009, while the prevalence of overweight (BMI ≥25 kg/m^2^) has more than tripled from 19.5 % in 1984 to 63.5 % in 2009 [11, 16, 22–25]. Accordingly, Libya was ranked 35^th^ on the list of the world's fattest countries based on this last estimation, in 2009 [26].

**Figure 1:**
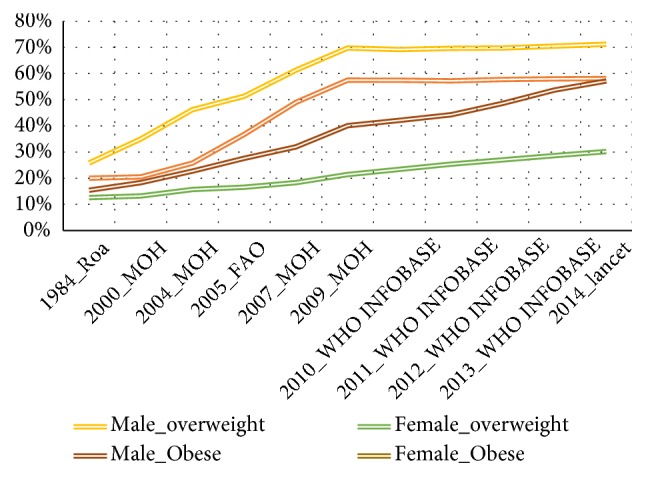
Trends of obesity and overweight among Libyan adults (male and female), in Libya (1984-2014).

**Figure 2:**
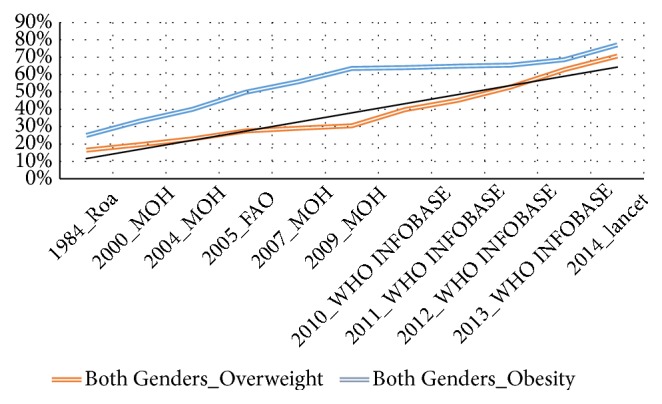
Trends of obesity and overweight (both genders), in Libya (1984-2014).

The prevalence of obesity and overweight in both genders has increased substantially from 1984 to 2014 [11, 16, 22–25]; see Figures 1 and 2. In addition, these figures have shown that prevalence of obesity and overweight is steadily increasing despite the fact that in recent years 2009-2014 relied on estimated prevalence instead of measuring the prevalence of obesity based on empirical studies. As a result of there not being any empirical studies after the Libyan national health survey (2009) [16, 24], the WHO heavily relied on the estimation of the prevalence of overweight and obesity, in the future consecutive years. Furthermore, given the increase of prevalence of obesity, the government would likely take action; however, in Libya, this is not the case, as a result of there not being sufficient evidence from empirical studies. Therefore, it is plausible that a study to monitor and describe secular trends in obesity among Libyan adults in order to promote the government in taking action on higher rates of obesity, by making early intervention a priority.

The Global Burden of Disease Study (GBD), coordinated by the Institute for Health Metrics and Evaluation (IHME), estimated the prevalence of obesity and overweight in Libyan adults at 71.9% in 2013 [27]. According to the estimation of the GBD and the IHME, Kuwait was ranked the fourth most obese country in the world, while Libya was ranked ninth in the list of the world's fattest countries [27–29]. This change in ranking from 35^th^ in 2011 to 9^th^ in 2013 may be attributable, at least in part, to the Libyan political revolution of 2011, which has resulted in an ongoing conflict among various radical militias, creating an unstable political, and unsafe environment that might influence both PA and access to healthy foods, which might eventually contribute to the obesity epidemic among the Libya population.

With respect to obesity-gender relationship, in most countries throughout the world, the prevalence of obesity is estimated to be greater in women than in men [30]; in three WHO regions, Africa, the EMR, and South East Asia, women were estimated to have roughly double the obesity prevalence of men [27–29]. Although obesity rates are higher among women than men in the Middle East and North Africa region (MENA), men obesity rates appear to be escalating more rapidly than women obesity rates [28, 29]. The scenario in Libya confirms this pattern because according to the last survey in Libya, in 2009, the prevalence of obesity in women was 40.1% compared to the 21.0% in [22, 24]. Concerning the obesity–age relationship, the causes of weight gain with ageing are indisputably multifactorial [31], as weight gain involves both biological and psychosocial components, both of which are important influences on body weight [32]. According to a comprehensive review study conducted by Musaiger (2011) [9], which concluded that, in most EMR countries, obesity increases more in ages up to 60, when obesity declines. In the Libyan context, despite Libyan belonging to the EMR, no studies have been conducted to investigate the obesity-age relationship.

## 2. Methods

### 2.1. Study Design and Setting

A quantitative approach in the form of cross-sectional study design was used to assess the prevalence of obesity among Libyan adults (male and female). This study employed the electoral register of the Benghazi Council as the sampling frame of which the research participants were drawn. The three consecutive strategies were applied to recruit the participants these include sampling parliamentary constituencies, polling districts, and finally, individuals.

**Figure 3:**
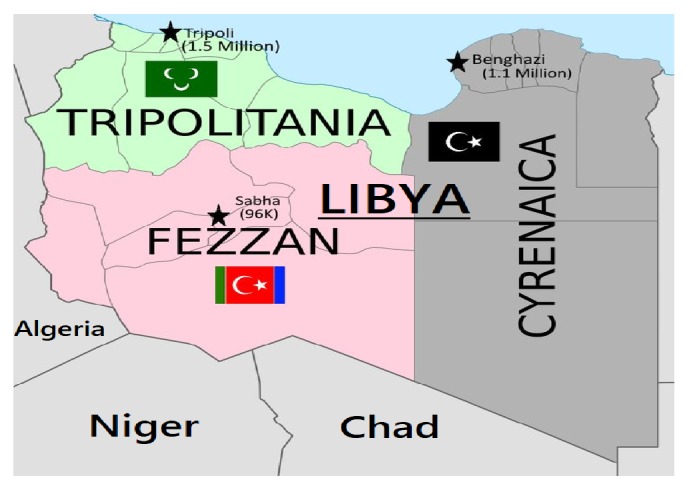
The map of Libya, including the three provinces (Cyrenaica, Tripolitania, and Fezzan).

Libya is considered three provinces (or states), Cyrenaica (Barqa or Benghazi) in the east, Tripolitania in the northwest, and Fezzan in the southwest [33, 34] (see Figure 3). Benghazi is the capital and largest city, the most populous city in Cyrenaica state. In recent years, the second largest city in Libya, Benghazi, has experienced a rapid increase in chronic diseases linked to obesity [17, 35]. Benghazi is an extremely wealthy economic city, characterised by urbanisation and modernisation in comparison to other Libyan cities. This makes Benghazi a suitable location for gathering data on this survey. According to the Libyan Bureau of Statistics and Census (BSC), 2012) [36], the population of Benghazi was 1.1 million inhabitants (one-fifth of the whole Libyan population). Given that Benghazi is a favourite destination for rural to urban migration, hence it becomes most likely to be ethnically diverse and multicultural. In addition, after the Libyan revolution in 2011 Benghazi has become a prominent destination for a monumental number of displaced Libyans, resettled in Benghazi, in search of safety, these include thousands of forces families loyal to the old regime (Tripolitania province) and a number of nomads who had fled from tribal conflicts in the desert (Fezzan province) [37, 38]. Therefore, sampling from this city would capture broad views of the Libyan population, of which in turn the gained results from the study can be generalised because the sample represents the whole population [35, 36].

### 2.2. Sampling Technique and Sample Size

A multistage cluster random sampling technique was justified and chosen as the sampling technique for this study in accordance with a highly recommended of some researchers or experts which agreed with conducting this sample over the geographical dispersal areas; this could be more efficient and economic [39–41]. This study has used the following Sample Size Formula. This study used the confidence level of 95%; therefore, the confidence interval (margin of error) = 5.

Sample Size (SS) = (1.96^2^(0.5)×(1  –  0.5))/0.05^2^ = 384 Libyan adults. Given the sample size of this study to be 384 Libyan adults and that the proposed response rate was predicted to be 75%, therefore, the final calculation for the sample size was 384/0.75 = 512 Libyan adults.

Sampling errors can be minimised by rigorous planning and consideration of the sampling technique. As with cluster sampling, the main weakness of multistage sampling is the possibility of lower accuracy than other probability sampling techniques. This is due to higher sampling errors than in simple random sampling since sampling errors can occur at any stage of multistage sampling [39, 42, 43]. However, sampling errors increase when there is a low number of the selected clusters, despite the differences between clusters, and it decreases when the homogeneity of cases per cluster is high [39–41]. Accordingly, the more clusters and participants this study recruits, the less likely this study is to experience sampling error. In order to minimise sampling errors and enhance the heterogeneity of the participants, this study selected 5 out of 11 clusters (parliamentary constituencies). Since the target population is dispersed widely across Benghazi, simple random sampling is unviable. Thus, on balance, multistage cluster sampling acquired the most relevance for the particular target population.

**Figure 4:**
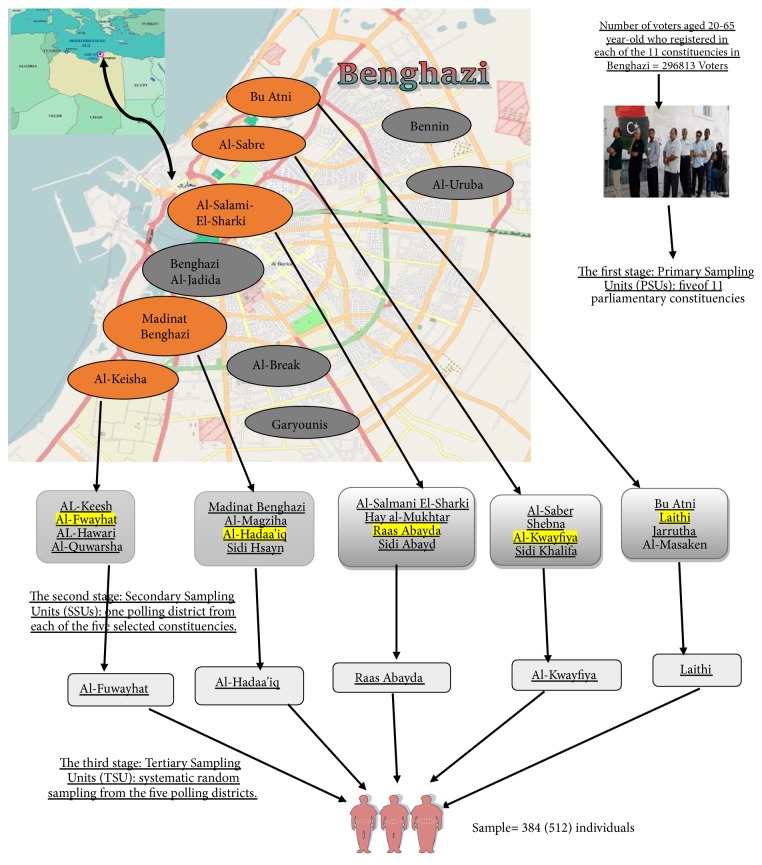
A schematic diagram of multistage cluster sampling.

A multistage cluster sampling in four stages was employed in this study to select a total sample of 512 participants. Prior to the first step, Cyrenaica (Benghazi) was selected as the setting of data collection by using a simple random sampling technique to select one of the three provinces (Cyrenaica, Tripolitania, and Fezzan) [33, 34]. The other three stages include the selection of the sample from sampling parliamentary constituencies, polling districts, and finally, individuals (see Figure 4). Benghazi's 11 parliamentary constituencies were used as the clusters to adhere with the Libyan High National Election Commissions' Regulations (HNEC) (2014) [44]. The 11 parliamentary constituencies are listed in alphabetical order from (A-Z) as follows: Al-Break, Al-Keisha, Al-Sabre, Al-Salami-El-Garb, Al-Salmani-ElSharki, Al- Uruba, Benghazi al-Jadida, Bu Alni, Benina, Garyounis, and Madinat Benghazi. In the first stage, Primary Sampling Units (PSUs) were obtained by the systematic random selection of 5 of the 11 constituencies that were listed alphabetically and were assigned serial numbers. These five were Al-Keisha; Al-Sabre; Al-Salmani-ElSharki; Bu Atni; and Madinat Benghazi. Each parliamentary constituency has three polling districts adhered to Benghazi Municipal Election (2012) [42, 45]. In the second stage, Secondary Sampling Units (SSUs) were derived by a simple random sampling of one polling district from each of the five constituencies (PSUs), specifically: Al-Fuwayhat; Al-Kwayfiya; Raas Abayda; Laithi; and Al-Hadaa'iq pertaining to Al-Keisha; Al-Sabre; Al-Salmani-ElSharki; Bu Atni; and Madinat Benghazi, respectively. Finally, in the third stage, ultimate or Tertiary Sampling Units (TSUs) were obtained by the systematic random sampling of potential participants, for sampling technique details; see Table 1.

### 2.3. Participants and Procedure

The research participants chosen for participating in this study have to meet the following criteria: they need to be aged 20-65 years; they speak the Arabic language; participants whose weight exceeded the capacity of the instrument (150kgs), which most previous studies failed to measure; legally eligible to register to vote. On the other hand, the exclusion criteria for participants were chair-bound; unstable on two feet; pregnant women; unable to stand upright; amputees; and a person who is subject to any legal incapacity to vote. In the Libyan context, due to impracticable data gathering methods include unreliable Internet services in many parts of Benghazi [46, 47] and the untrusted postal service along with the insecurity and instability in Libya's current situation [48, 49], therefore, the feasible method using a questionnaire administered under personal supervision of the researcher to the respondents. The study involved exactly 401 visits to Libyan adults across the five polling districts in Benghazi. The meeting places with participants were varied, ranging from the participants' homes to their nearest health facilities, or a meeting at a mutually agreeable place if requested by the participants. Most females favoured meeting at their nearest health facilities, where they live, as they may feel more comfortable with this. The research team contacted the participants in advance to inform the participants about the pending survey via phone calls.

This study was able to assign a team of qualified male and female nurses in each district in line with an approval granted by the health sector in Benghazi, for taking the anthropometric measurements for all the participants. Religious and cultural barriers often force Libyan women to have a negative attitude towards being cared for by male nurses; thereby, this study allocated male and female nurses to overcome this issue. Finally, the data collection for this main study took place from Augusts to December 2015.

### 2.4. Measuring Obesity

Despite several anthropometric measuring tools for assessing obesity risk, mainly those termed ‘field methods' include BMI, waist circumference (WC), waist-to-hip ratio (WHR), and waist-to-height ratio (WHtR), BMI remains the most widely used tool for measuring the prevalence of obesity at the population level in extensive epidemiological research studies [50]. BMI is specified as weight in kilograms divided by height in meters squared [51]. Although BMI has been considered the most accepted and adopted method for assessing body composition worldwide [52], however, there are various limitations of BMI which are represented in factors; for instance, age, sex, ethnicity, puberty, and bone and muscle mass can influence the relationship between BMI and body fat, resulting in distorted readings [50, 53, 54]. Using BMI alone as a tool for assessing weight status is an unfeasible indicator; therefore, experts have suggested that two methods must be paired with BMI, for instance, WC or WHR [51]. Thus, in order to minimise limitations with respect to using the BMI alone in this study, with Visceral Fat Level, were measured in conjunction with a BMI.

The World Health Organisation (WHO) [49] and Centre for Disease Control and Prevention (CDC) [55] categorise underweight, overweight, and obesity in adults based on different cut-off values; see Table 2 for details.

### 2.5. Anthropometric Measurements

The anthropometric measurements were taken from the participants in this study by the qualified nurses. Two pieces of portable equipment were employed, including a portable audiometer (to measure height (cm) to the nearest 0.1 centimetres) and Tanita BC-601 Segmental Body Composition Monitor (to determine the following measurements: Percent Body Fat nearest to 0.1%, weight to the nearest 0.1 kilograms, BMI (Kg/M^2^), and Visceral Fat Level). The Segmental Body Composition Monitor is likely to eliminate the restriction of using BMI alone because it provides two coupled measurements BMI and Visceral Fat Level.

### 2.6. Questionnaire Design

This study adopted and used a preexisting questionnaire which is WHO STEPS Instrument for Noncommunicable Diseases Risk Factor Surveillance (STEPS-NCDRFS) [56], which consists of two steps; Step 1, demographic information and behavioural measurements, and Step 2, physical measurements. Regarding Step 1, information on sociodemographic data was adopted to investigate about the participants' sociodemographic characteristics (age, gender, marital status, and ethnicity) as well as their socioeconomic status (SES) characteristics (employment status, income, and educational level). Apart from Step 1, the sociodemographic data and SES characteristics of the WHO STEPS Instrument survey, this study disregarded the other scales of the same questionnaire, mainly behavioural NCD risk factors (including the sections of dietary intake, alcohol consumption, smoking status, physical activities, and sedentary behaviour) because this study has adopted the other three preexisting questionnaires which addressed these themes (which were rigorously relative to the study). With respect to Step 2, anthropometric measurements, including Height (cm), Body Mass Index (BMI) (Kg/M^2^), Percent Body Fat (%), and Visceral Fat Level, are ranging from 1 to 59.

Three Libyan certified translators were allocated to achieve the forward-backwards-forward translation technique for the questionnaire, and they agreed on the final version of it to be tested. To increase the validity and reliability of this tool Pearson's correlation coefficient was performed to test for correlations between the score for each item of the questionnaire and the total score (r=0.521, p<.001). The pretest data were also tested for internal consistency using Cronbach's alpha (*α* = 0.763).

### 2.7. Statistical Analysis Methods

Statistical Software Package for Social Sciences (SPSS, version 24.0) was used to enter numeric data and perform analysis. Descriptive statistics were performed to describe the participants' characteristics in relation to sociodemographic and SES characteristics. In addition, a cross-tabulation analysis was used to describe the relationships between age, gender, and BMI. Two models of BMI categories were employed in this study. First pattern: BMI was used as a continuous variable in order to compute the mean BMI among Libyan men and women. Second pattern: BMI was recoded into three categories as follows: normal weight (18.50-24.99), overweight (25.00 ≤ BMI < 30.00), and obese (I, II, III: ≥30.00). The second pattern was used to estimate the prevalence of overweight and obesity among Libyan men and women.

### 2.8. Ethical Considerations

The following three bodies have granted ethical approval for this study: Bedfordshire University, UK (IHREC/303, dated 28 January 2014), Omar Al-Mukhtar University, Libya (UOA/44, dated 29 January 2014), and Ministry of Regional Health, Benghazi, Libya (MORHB/ 24, dated 30 January 2014). All participants were entirely informed of all aspects of the study. In addition, written, signed, and dated informed consent forms were obtained from all participants before they agreed to participate in a survey.

## 3. Results

### 3.1. The Recruitment Outcome

Table 3 shows the outcome of the recruitment of the research participants in each of the polling districts for this study. It depicts the total numbers of ineligible participants 35 (7%); participants who opted out 24 (4.5 %); participants who were not approached 49 (10%); and those who did not complete questionnaires 3 (.5%). The total number of participants who participated in this study was 401 (78%) (148 (29%) males and 253 (49%) females).

### 3.2. Response Rates

Table 4 illustrates that the actual response rate achieved in the whole study was 78%, which was slightly higher than the predictable response rate (75%). The table also shows that the response rate was achieved in each polling district ranged from 72%, in Al-Fuwayhat and RaasAbayda, to 82%, in Laithi.

### 3.3. Demographic Characteristics of the Sample

Table 5 demonstrates that 401 Libyan adults aged 20-65 years took part in this study; 253 were female (63.1%); the highest proportions of participants were aged from 40 to 49 years (29 %). The majority of respondents were married (67.1%). Regarding ethnic groups; 84.6% were Arabic, 10.7% were Berbers ‘Imazighen', and 4.7% were Toubou. Concerning the participants, educational level 51.4% of all participants (male and female) had a high level of education; 77.6% of all of the participants were employed, and 34% and 42% of all participants reported that they earned a moderate and high income, respectively.

**Figure 5:**
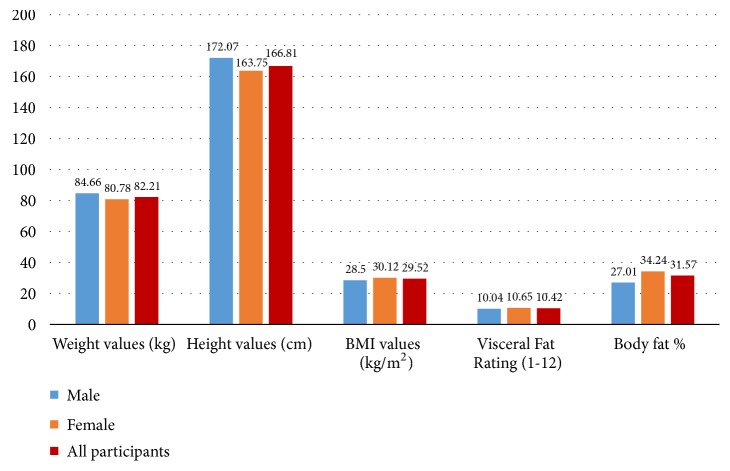
Mean of anthropometric measurements in Libyan adult population.

Figure 5 demonstrates the means of anthropometric measurements for all participants as follows: the mean BMI was 29.52 (+/- 6.19) kg/m^2^, the participants' mean visceral fat was 10.42 (+/-4.1), and the mean body fat percentage was 31.57 (+/-9.42%). Additionally, the figure shows that males had a higher physical measurements values than females in terms of the mean weight which was 84.66 (+/-17.50) kg and the mean height which was 172.06 (+/- 7.68) cm for males. In contrast, females had higher anthropometric measurement values than males about the mean BMI was 30.12 (+/- 6.54) kg/m^2^, mean visceral fat was 10.65 (+/- 4.2), and mean body fat percentage was 34.24 (+/- 9.51%) for females.

Table 6 demonstrates that the prevalence of obesity among adults was 42.4%, whereas the prevalence of being overweight and being of normal weight was 32.9% and 24.7%, respectively. In addition, the prevalence of being overweight and obese was 75.3%. Among men, the prevalence of obesity was 33.8% and overweight 32.4%, while among women, the prevalence of obesity was 47.4% and overweight 33.2%. Interestingly, the result obtained from this survey shows that there are no underweight participants.

**Figure 6:**
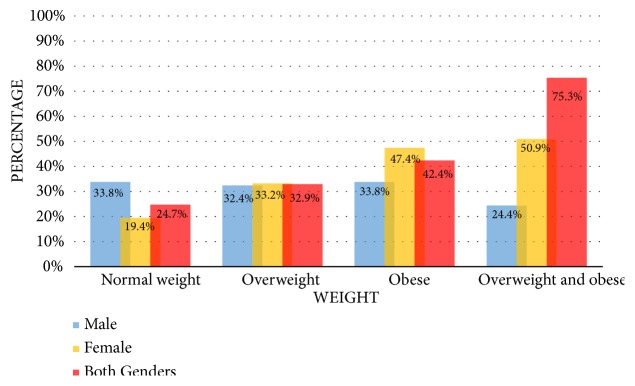
The prevalence of normal weight, overweight, and obesity among Libyan adults (male and female).

Figure 6 shows the prevalence of normal weight; overweight; obesity; and both overweight and obesity among Libyan adults (male and female).

**Figure 7:**
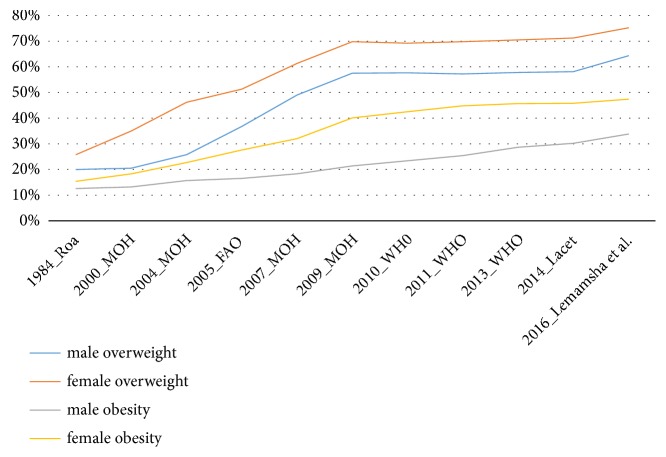
Trends of obesity and overweight among Libyan adults (male and female), in Libya (1984-2016).

**Figure 8:**
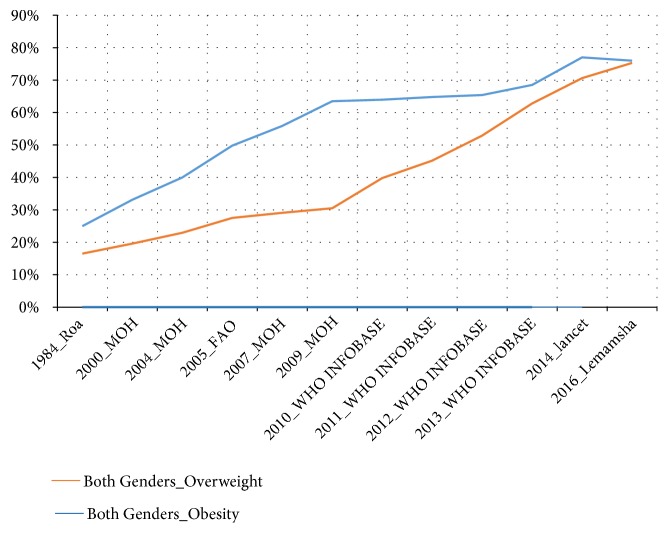
Trends of obesity and overweight (both genders), in Libya (1984-2016).

Figures 7 and 8 show trends of obesity and overweight among Libyan adults (male, female, and both genders), in Libya (1984-2016), which included the outcome of the prevalence of overweight and obesity revealed by this study (Lemamsha et al., 2016)

Table 7 shows that, for females, the highest obesity rate was in middle-aged adults, aged 40-49 years, while the second highest rate was in older adults, aged 60-65 years. In contrast, the highest obesity rate for males was in older adults aged 60-65 years, while the lowest rate was in younger adults, aged 20-29 years, as for females.

**Figure 9:**
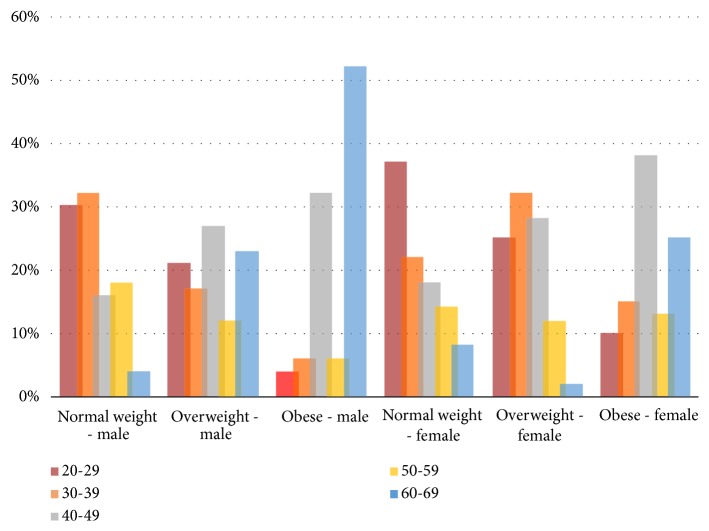
Age groups of the participants and the prevalence of normal weight, overweight, and obesity among Libyan adults (male and female).

Figure 9 shows the difference between age groups of the participants and the prevalence of normal weight, overweight, and obesity among Libyan adults (male and female).

## 4. Discussion

In this cross-sectional study which aimed to estimate and describe the prevalence of overweight and obesity among Libyan men and women, as 401 Libyan adults were recruited in the study, the actual response rate achieved was 78%, which was rated “very good” (70-85%) in accordance with the classification of response-rate bands [37]. This study revealed that the prevalence of obesity among Libyan adults was 42.4%, whereas the prevalence of being overweight was 32.9%. In addition, the prevalence of obesity among women was significantly higher than that among men (47.4% vs 33.8%). Although an increase in the prevalence of obesity has been reported in many countries around the world, the rate of increase in Libya has been observed to be particularly high. The prevalence of obesity in Libya has more than doubled in the last three decades, from 12.6% in 1984 to 30.5% in 2009 [11, 16, 22–25]. Consequently, it may be concluded that the prevalence of obesity in Libya had increased by more than a third over the previous six years from 2009 when it was 30.5% until this year 2016 when it was estimated to be 42.4% in this study. Since the present study adopted the Segmental Body Composition Analyser to identify anthropometric measurement readings, which were conducted by highly trained and experienced nurses, the results of this study are arguably more accurate and reliable than those of other studies in the Arab region, which adopted self-administered questionnaires that included self-reported anthropometric measures [57–59].

One possible explanation for the high prevalence of obesity among Libyans adults is the deterioration in the current political situation in Libya, including an unsafe physical environment due to battles among militias coupled with weak Libyan government performance in upholding the rule of law. Thereby this has resulted in their inability to protect civilians from rights abuses in Benghazi [60, 61]. Hence, Libyans tend to stay at home most of the day, eating unhealthy foods, which are the ‘staple food commodities' of the country, subsidised by the Libyan government which makes them widely available to the general public. Other possible clarifications are that this hazardous environment is currently [2016] having an impact on Libyans' daily activities, for example, encouraging citizens to remain indoors for extended periods. This increased level of inactivity has likely exacerbated the epidemic of obesity in Libya. Further possible explanations are the absence of physical activities due to a curfew enforced by the government [58, 59]; deteriorating Libyan health system performance, resulting in Libya's healthcare facilities become completely ineffectiveness in preventing and controlling obesity among Libyans and in spreading health awareness among Libyan population about obesity [11–13, 62], whether by the old or the new regime. Collectively, these factors may contribute to an exacerbation of the obesity epidemic; therefore, it is arguable that the high prevalence rate of obesity revealed by this study is unsurprising.

The Global Burden of Disease Study (GBD), coordinated by the Institute for Health Metrics and Evaluation (IHME) [27], estimated the prevalence of obesity and overweight in Libyan adults at 73.9% in 2013 (43.9% for obesity and 30.0 % for being overweight), whereas this study found that the prevalence of obesity and overweight is at 75.3% in 2016 (42.4% for obesity and 32.9% for being overweight). Thus, the prevalence of obesity and overweight in Libya estimated by the GBD study (71.9%) is slightly lower than the prevalence of obesity and overweight in Libyan adults estimated by this study in 2016 (75.3%).

While this study found that Libya has a prevalence rate of 42.4% for obesity, 32.9% for being overweight, and 75.3% for both, it found BMI figures of 28.50 (+/- 5.40) for men and 30.12 (+/- 6.54) kg/m^2^ for women. In comparison with, Nauru is the world's fattest country, where the obesity rate is 71.7%, with an average BMI of 34 to 35 kg/m^2^. Afterwards, it is followed by Samoa, the Federated States of Micronesia, American Samoa, and several Arab countries of the Middle East [22, 27, 63]. Conversely, Bangladesh is the world's thinnest nation, with an average BMI of 20.5 kg/m^2^ for women and 20.4 kg/m^2^ for men [22, 27, 64].

Currently, the prevalence of obesity in five of the Arab Gulf countries rivals and exceeds that of the US. The World Obesity Federation has estimated that the highest prevalence of obesity in five of the Arab Gulf countries was found to be among adults in Kuwait [26]. Conversely, the lowest prevalence of obesity was found to be in the United Arab Emirates (UAE) [20, 65] These prevalence rates have enabled the Arab Gulf countries to rival and occupy the ‘top ten' most obese countries in the world [27, 29]. Libya was ranked ninth in the list of the world's fattest countries, based on the classification presented by a comprehensive review conducted by Ng et al. (2014) [27]. However, the more up-to-date calculations and findings from this study may necessitate a reclassification of the fattest countries in the world. With these latest figures of the prevalence of obesity for Libya (33.8% of men; 47.4% of women) pushing this country further up the list, to occupy a place between Qatar (44% of men; 54.7% of women) and Saudi Arabia (30% of men; 44.4% of women), Libya should in fact occupy sixth place, which would be a rise of three places on the list according to the estimation of the Global Burden of Disease Study [30].

The finding of this study concerning the gender-obesity relationship shows that although explanations for the gender variance of obesity prevalence have been discussed and debated widely, most explanations which were argued pertain to developing countries rather than to developed counties. In the Arab region, Arab women are less likely to take time to exercise due to religious and culture barriers. Barriers that such women face in engaging in physical activities include a lack of time, parenting demands, and gender stereotyping whereby child-rearing and domestic chores are considered to be ‘women's work' [9, 66]. Throughout both North and sub-Saharan Africa, for example, obesity and physical inactivity among both genders carry connotations of high social prestige, fertility, good health, and affluence. Moreover, gender differences in the cultural (regional) dress may intensify gender differences in obesity; for example, women who wear a long, traditional loose-fitting dress may be hiding their body-shape regarding “hidden obesity” or the converse phenomenon of “hidden hunger” [67]. Cultural restrictions among North and sub-Saharan African women and sociocultural beliefs common in the EMR may indirectly deter women from engaging in leisure-time physical activity and thus may lead to weight gain among women in these regions more than among men. In conservative societies, such as in the MENA, women are often overprotected because of cultural or religious barriers [9, 68].

The finding of this study concerning the age-obesity relationship is consistent with those of previous studies which found a significant association between obesity and age [69, 70]. In addition, the finding of this study is similar to those of a comprehensive review study conducted by Musaiger (2011) [9], which concluded that, in most EMR countries, obesity increases with age up to 60 years of age, when obesity starts to decline in both men and women. One possible explanation for middle-aged (aged 40-49 years) women having higher rates of obesity than women from other age groups is multiparity. In contrast, it was older men (aged 60-65 years) who have higher rates of obesity than men from other age groups do. One explanation could be retirement, which is usually accompanied by behavioural changes including unhealthy food intake; less structured meal times or a change in eating patterns; and becoming less physically active, for example, due to no longer getting up early to travel to work on a daily basis [71]. It is agreed that the slowing metabolism that occurs at this age also promotes changes in body composition, favouring increased body weight [72]. Furthermore, since the second highest obesity rate was in women aged 60-65 years, it is likely that biological changes such as menopause are responsible for weight gain in this age group due to a decline in women's oestrogen levels, which probably affects the fat distribution and may increase the risk of obesity [73].

## 5. Limitations

The first limitation is that the cross-sectional design was unable to draw directional causal inferences because it is signified as a ‘snapshot' study, which in turn might produce contradictory results if alternative timeframe had been chosen to repeat the survey. With respect to limitation of Benghazi electoral register ‘a sampling frame' was that certain groups in the society were excluded as they had been prohibited from voting according to Libyan Electoral Law 2012, Part III: The Right to Vote under article number four [45]. These groups are any person determined legally to be incompetent, with mental illness; those working in the police force, or as judges, as well as those convicted of crimes stipulated in the penal code [74]. Excluded groups in this study represented only 7 % of the whole of the target population, that is, only a minor proportion of the Libyan population. It can be concluded that the results are still representative of the adult's Libyan population and can, therefore, be generalised. Nevertheless, it is prudent to consider the exclusion of certain groups as one of the limitations of this study. In addition, using a portable Tanita BC-601 Segmental Body Composition Monitor is unable to provide BMI readings for the following groups: pregnant women; amputees; those unsteady on their feet, too frail or unable to stand upright. Excluding the aforementioned groups due to practical reasons, it is arguably another limitation of this study.

## 6. Practical and Policy Implications

The study might encourage other researchers in Libya or other Arab countries to follow and carry out the same trajectory in order to contribute to information about subject response rates in the Arab region; to date, there is a lack of relevant information about response rates, and further studies are required. The mean values of anthropometric measurements obtained in this study such as weight, height, visceral fat, body fat percentage, and BMI could be used as indicators for the Libyan population in comparative research with other developing and developed countries, while the mean values for visceral fat and body fat percentage—both outcome variables analogous to BMI—could be used to test in association with predictor variables. These findings could be useful to researchers in diverse aspects of health-related research, particularly related to heart disease and diabetes. This study has contributed to the standard list of exclusion criteria recommended by obesity experts through reducing the list in order to increase the chances of recruiting as many research participants as possible, including those who weigh more than 130kgs. On the other hand, it added one new exclusion criterion: participants who are amputees, because such cases require a special formula to calculate BMI, adapted to account for the estimated weight of the missing limb [75].

## 7. Conclusion

The findings of this study have revealed that overweight and obesity are one of the fastest growing, and it continues to be a significant public health crisis facing the Libyan government. Regarding the gender-obesity relationship, this study showed steadily that overweight and obesity rates are higher among Libyan women than Libyan men due to the absence of prevention and control measures in Libya. With respect to the relationship between rates of obesity, age, and gender this study found that the prevalence of obesity in both Libyan men and women increases with age since the findings revealed that the highest rate of excess weight for males and the second highest rate of excess weight for females were in older adults aged 60–65 years. Further studies are required to assert the generalisability of the results include Libyan national- representative surveys are needed to collect more data to monitor and to describe secular trends in the prevalence of overweight and obesity in Tripolitania and Fezzan Libyan provinces across different genders and age groups. These findings could inform Libyan health authorities with regard to policies and interventions that are urgently needed for preventing or controlling the obesity epidemic in Libya.

## Figures and Tables

**Table 1:** Sampling technique.

S.N	The five selected constituencies	The five selected polling district	Number of the voters/polling district	% of sample size/polling district	Total hypothesised Sample size	Total sample size
1.	Al-Salmani-ElSharki	Raas Abayda	8480	15%	75	54

2.	Al-Keisha	Al-Fuwayhat	8012	14%	71	51

3.	Al-Sabre	Al-Kwayfiya	10433	18%	92	75

4.	Madinat Benghazi	Al-Hadaa'iq	19102	33%	169	135

5.	Bu Atni	Laithi	11858	20%	105	86

Total			57885	100 %	512	401

**Table 2:** The international classification of body weight in adults.

Classification	Principle BMI cut off point (kg/m2)	Risk of co-morbidities
Underweight	<18.5	Low^a^
Healthy weight	18.5–24.9	Average
Overweight	25–29.9	Increased
Obesity, class I	30–34.9	Moderate
Obesity, class II	35–39.9	Severe
Obesity, class III	≥40-	Very severe

^*∗*^Other health hazards may be associated with low BMI. Source: adapted from WHO, 2006 [49].

**Table 3:** The outcome of the recruitment of the participants in each of the five polling districts for this study (n=401).

Names of polling districts	Number of Ineligible participants	Number of the participants who opted out	Number of the participants who not approached	Incomplete surveys	Completed surveys			Total of the prospective participants
					Male	Female	Total	
Al-Fuwayhat	5	5	9	1	21	30	51	71

Al-Kwayfiya	4	4	8	1	28	47	75	92

Raas Abayda:	7	3	10	1	20	34	54	75

Laithi	7	2	10	Nil	37	49	86	105

Al-Hadaa'iq	12	10	12	Nil	42	93	135	169

Total N (%)	35 (7%)	24 (4.5 %)	49 (10%)	3 (5%)	148 (29%)	253 (49%)	401 (78%)	512

**Table 4:** The response rate in each polling district and the total response rate for the whole study.

Polling district	Response rate achieved in
	each polling district
Al-Fuwayhat	51/71 × 100 = 72%
Al-Kwayfiya	= 75/92 × 100 = 81.5 = 82%
Raas Abayda:	= 54/75 × 100 = 72%.
Laithi	= 86/105 × 100 = 82%.
Al-Hadaa'iq	135/169 × 100 = 80%.
The total response rate of this study	401/512 × 100 = 78%

**Table 5:** Sociodemographic characteristics and anthropometric measurements in Libyan adult population vary by gender.

Demographic and Socio-Economic Characteristics	Total	Male (M)	Female (F)
	N (%)	N (%)	N (%)
Gender	401 (100)	148 (37)	253 (63)

Age			
20 – 29	78 (19)	27 (18)	51 (20)
30 – 39	83 (21)	27 (18)	56 (22)
40 – 49	115 (29)	37 (25)	78 (31)
50 – 59	50 (12)	18 (12)	32 (13)
60 – 65	75 (19)	39 (26)	36 (14)

Racial group			
Arabic	339 (84.6)	129 (87.2)	210 (83)
Berbers ‘Imazighen'	43 (10.7)	15 (10.1)	28 (11.1)
Toubou	19 (4.7)	4 (2.7)	15 (5.9)

Marital Status			
Single (Unmarried)	132 (32.9)	47 (31.8)	85 (33.6)
Married^★^	269 (67.1)	101 (68.2)	168 (66.4)

Level of education:			
Low educational level *∗*	77 (19.2)	32 (21.6)	45 (17.8)
Moderate educational level*∗∗*	118 (29.4)	41 (27.7)	77 (30.4)
High educational level *∗∗∗*	206 (51.4)	75 (50.7)	131 (51.8)

Monthly Income: “ (LYD)” *∗*			
Low income < 1999	95 (24)	9 (6)	86 (34)
Moderate income 2000–2999	135 (34)	45 (30)	90(36)
High income: 3000–3999	171 (42)	94 (64)	77 (30)

Occupation:			
Employed groups ☆	311 (77.6)	120 (81.1)	191 (75.5)
Unemployed groups ☆☆	90 (22.4)	28 (18.9)	62 (24.5)

Physical	All participants	Male	Female
Anthropometric	(N= 401)	(N= 148)	(N= 253)
characteristics	“Mean (+/- SD)”	“Mean (+/- SD)”	“Mean (+/- SD)”

Weight values (kg)	82.21 (+/- 17.47)	84.66 (+/- 17.50)	80.78 (+/- 17.33)

Height values (cm)	166.81 (+/- 9.15)	172.07 (+/- 7.69)	163.75 (+/- 8.54)

BMI values (kg/m^2^)	29.52 (+/- 6.19)	28.50 (+/- 5.40)	30.12 (+/-6.54)

Visceral Fat Rating (1-12)	10.42 (+/- 4.1)	10.04 (+/- 3.9)	10.65 (+/-4.2)

Body fat %	31.57 (+/- 9.42)	27.01 (+/- 7.31)	34.24 (+/-9.51)

^★^ *Married*: “being married; divorced separated; widowed.”

**∗** *Low educational level*: “no formal schooling; less than a primary school; primary school completed.”

**∗** **∗** *Moderate educational level*: “secondary school completed; high school completed.”

**∗** **∗** **∗** *High educational level*: “college/university completed; postgraduate degree.”

☆*Employed groups*: “government employee; nongovernment employee; self-employed; nonpaid & student.” ☆☆*Unemployed groups*: “housework; retired; unemployed (able to work); and unemployed (unable to work).

**∗** *Libyan Dinar (LYD)* = 1/2 Pound under current exchange rate.

**Table 6:** The prevalence of normal weight, overweight, and obesity amongst Libyan adults (male and female).

Three weight status categories (BMI categories)	Gender		Prevalence of overweight and obesity in Libyan adults
	Male	Female	
	N (%)	N (%)	
Normal weight	50 (33.8)	49 (19.4)	99 (24.7)
(BMI=18.5–24.9 kg/m^2^)			

Overweight	48 (32.4)	84 (33.2)	132 (32.9)
(BMI= 25–29.9 kg/m^2^)			

Obese	50 (33.8)	120 (47.4)	170 (42.4)
(BMI ≥ 30 kg/m^2^)			

Overweight & obese	98 (24.4)	204 (50.9)	302 (75.3)
(BMI ≥ 25 kg/m^2^)			

**Table 7:** Age groups of the participants and the prevalence of normal weight, overweight, and obesity amongst Libyan adults (male and female).

Age	Males			Females		
	Normal weight (BMI=18.5–24.9) N (%)	Overweight (BMI=25–29.9) N (%)	Obesity (BMI *⩾* 30) N (%)	Normal weight (BMI=18.5–24.9) N (%)	Overweight (BMI=25–29.9) N (%)	Obesity (BMI *⩾* 30) N (%)
20 – 29	15 (30)	10 (21)	2 (4)	18 (37)	21 (25)	12 (10)

30 – 39	16 (32)	8 (17)	3 (6)	11 (22)	27 (32)	18 (15)

40 –49	8 (16)	13 (27)	16 (32)	9 (18)	24 (28)	45 (38)

50 – 59	9 (18)	6 (12)	3 (6)	7 (14.3)	10 (12)	15 (13)

60– 65	2 (4)	11 (23)	26 (52)	4 (8.2)	2 (2)	30 (25)

## Data Availability

The data used to support the findings of this study are available from the corresponding author upon request.

## Abbreviations

BMI: Body Mass Index
WC: Waist circumference
WHR: Waist-to-hip ratio
WHtR: Waist-to-height ratio
BIA: Bioelectrical impedance analysis
GBD: The Global Burden of Disease Study
NOO: National Obesity Observatory
AACE: The American Association of Clinical Endocrinologists.

## References

[1] Guh D. P., Zhang W., Bansback N., Amarsi Z., Birmingham C. L., Anis A. H. (2009). The incidence of co-morbidities related to obesity and overweight: A systematic review and meta-analysis. *BMC Public Health*.

[2] Lenz M., Richter T., Mühlhauser I. (2009). The morbidity and mortality associated with overweight and obesity in adulthood: A systematic review. *Deutsches Ärzteblatt International*.

[3] World health organisation (WHO) (2018). *Obesity and Overweight*.

[4] Medvedyuk S., Ali A., Raphael D. (2017). Ideology, obesity and the social determinants of health: a critical analysis of the obesity and health relationship. *Critical Public Health*.

[5] Nyberg S. T., Batty G. D., Pentti J. (2018). Obesity and loss of disease-free years owing to major non-communicable diseases: a multicohort study. *The Lancet Public Health*.

[6] GBD 2015 Obesity Collaborators (2017). Health effects of overweight and obesity in 195 countries over 25 years. *The New England Journal of Medicine*.

[7] Romain A. J., Marleau J., Baillot A. (2018). Impact of obesity and mood disorders on physical comorbidities, psychological well-being, health behaviours and use of health services. *Journal of Affective Disorders*.

[8] World Obesity Federation (WOF) (2018). *World Obesity Day*.

[9] Musaiger A. O. (2011). Overweight and obesity in Eastern Mediterranean Region: Prevalence and possible causes. *Journal of Obesity*.

[10] Finucane M. M., Stevens G. A., Cowan M. J. (2011). National, regional, and global trends in body-mass index since 1980: systematic analysis of health examination surveys and epidemiological studies with 960 country-years and 9·1 million participants. *The Lancet*.

[11] Lemamsha H., Papadopoulos C., Randhawa G. (2018). Understanding the risk and protective factors associated with obesity amongst Libyan adults - A qualitative study. *BMC Public Health*.

[12] Lemamsha H., Papadopoulos C., Randhawa G. (2018). Perceived environmental factors associated with obesity in libyan men and women. *International Journal of Environmental Research and Public Health*.

[13] Lemamsha H. Exploring the risk and protective factors associated with obesity amongst Libyan adults (20-65 years).

[14] Altajori N. N., Elshrek Y. M. (2017). Review article: risk factors for non-communicable diseases in Libya. *The Egyptian Journal of Hospital Medicine*.

[15] Abdul Rahim H. F., Sibai A., Khader Y. (2014). Non-communicable diseases in the Arab world. *The Lancet*.

[16] World Health Organization (WHO) (2009). *Libya STEPS Survey 2009*.

[17] World Health Organization (WHO) *Noncommunicable Diseases (NCD) Country Profiles: Libya*.

[18] World Health Organization (WHO) *Humanitarian Crisis in Libya: Public Health Risk Assessment And Interventions*.

[19] World Health Organization (WHO) (2018). *Libya Health Emergencies and Humanitarian Update*.

[20] Libyan Organization Of Policies & Strategies (LOOPS) *The Health Sector in Libya: Situation and Challenges*.

[21] El-Fallah M. The development of the Libyan health system to improve the quality of health services.

[22] World Obesity Federation (WOF) (formerly IASO) *World Map of Obesity*.

[23] Elmehdawi R. R., Albarsha A. M. (2012). Obesity in Libya: a review. *Libyan Journal of Medicine*.

[24] Ministry of Health (MOH) (2010). *Libya STEPS Noncommunicable Disease Risk Factors Survey*.

[25] Rao G. M., Morghom L. (1984). Relationship of obesity to diabetes. *Hormone and Metabolic Research*.

[26] Central Intelligence Agency (CIA) World Fact book: *Obesity - Adult Prevalence Rate Gives the Percent of a Country's Population Considered Obese*.

[27] Ng M., Fleming T., Robinson M., et al (2014). Global, regional, and national prevalence of overweight and obesity in children and adults during 1980–2013: a systematic analysis for the Global Burden of Disease Study 2013. *The Lancet*.

[28] World Health Organization (WHO) *Obesity: Situation and Trends*.

[29] Harvard School of Public Health (2012). *Adult Obesity*.

[30] Garawi F., Devries K., Thorogood N., Uauy R. (2014). Global differences between women and men in the prevalence of obesity: is there an association with gender inequality?. *European Journal of Clinical Nutrition*.

[31] Grundy S. M. (1998). Multifactorial causation of obesity: implications for prevention. *American Journal of Clinical Nutrition*.

[32] Sobal J. (2001). Commentary: Globalization and the epidemiology of obesity. *International Journal of Epidemiology*.

[33] Karim M. (2018). State of Libya. *The Government and Politics of the Middle East and North Africa*.

[34] Bruce R. (2015). *Libya: Continuity and Change*.

[35] Beshyah S. (2010). Non-communicable diseases and diabetes care guidelines: epidemiology and call for collective action. February, 6th 2010, dat elmad conference hall complex, Tripoli, Libya. *Ibnosina Journal of Medicine and Biomedical Sciences*.

[36] Libyan Bureau of Statistics and Census (LBSC) (2012). *National Statistics: The Final Results of Census for Libya*.

[37] Bredeloup S., Pliez O. (2011). The Libyan Migration Corridor. *Research Report Case Study, EU-US Immigration Systems*.

[38] Carboni A., Moody J. (2018). Between the cracks: actor fragmentation and local conflict systems in the libyan civil war. *Small Wars and Insurgencies*.

[39] Bryman A. (2016). *Social Research Methods*.

[40] Nouri F., Feizi A., Mohammadifard N., Sarrafzadegan N. (2018). Methods of sampling and sample size determination of a comprehensive integrated community-based interventional trial: Isfahan Healthy Heart Program. *ARYA Atherosclerosis*.

[41] Spittal M. J., Carlin J. B., Currier D. (2016). The Australian longitudinal study on male health sampling design and survey weighting: implications for analysis and interpretation of clustered data. *BMC Public Health*.

[42] Daniel J. (2011). *Sampling Essentials: Practical Guidelines for Making Sampling Choices*.

[43] Shackman G. (2001). *Sample Size And Design Effect*.

[44] Libyan’s High National Election Commission (HNEC) (2012). *Libyan Electoral Law*.

[45] Libyan’s High National Election Commission (HNEC) Constitutional Drafting Assembly: Processing of voter registration. http://hnec.ly/en/?page_id=7496#.

[46] Elareshi M. (2013). *News Consumption in Libya: A Study of University Students*.

[47] Elzawi A., Wade S., Kenan T., Pislaru C. internet and emerging information technologies in libyan universities into reduce digital divide.

[48] Kennard C. (2001). Getting our Journal to developing countries. *Journal of Neurology, Neurosurgery & Psychiatry*.

[49] Selvik K., Utvik B. O. (2015). *Oil States in the New Middle East: Uprisings and Stability*.

[50] Bhurosy T., Jeewon R. (2013). Pitfalls of using body mass index (BMI) in assessment of obesity risk. *Current Research in Nutrition and Food Science*.

[51] World Health Organization (WHO) (2006). *WHO Body Mass Index (BMI) Classification*.

[52] Harvard School of Public Health (2019). *Measuring Obesity*.

[53] Centers for Disease Control and Prevention (CDC) Body Mass Index: Considerations for Practitioners. https://www.cdc.gov/obesity/downloads/bmiforpactitioners.pdf.

[54] Public Health England Measurement of Obesity. http://www.noo.org.uk/NOO_about_obesity/measurement.

[55] Centers for Disease Control and Prevention (CDC) About Adult BMI | Healthy Weight | CDC. https://www.cdc.gov/healthyweight/assessing/bmi/adult_bmi/.

[56] World Health Organization (WHO) WHO STEPS Instrument for Noncommunicable Disease Risk Factor Surveillance. https://www.who.int/ncds/surveillance/steps/instrument/steps_instrument_v3.1.pdf.

[57] Dijkshoorn H., Ujcic-Voortman J. K., Viet L., Verhoeff A. P., Uitenbroek D. G. (2011). Ethnic variation in validity of the estimated obesity prevalence using self-reported weight and height measurements. *BMC Public Health*.

[58] Martins P. C., De Carvalho B. M., Machado C. J. (2015). Use of Self-Reported measures of height, Weight and body mass index in a rural population of northeast Brazil. *Revista Brasileira de Epidemiologia*.

[59] Bowring A. L., Peeters A., Freak-Poli R., Lim M. S. C., Gouillou M., Hellard M. (2012). Measuring the accuracy of self-reported height and weight in a community-based sample of young people. *BMC Medical Research Methodology*.

[60] Chivvis C., Martini J. (2014). *Libya After Qaddafi: Lessons and Implications for the Future*.

[61] Tabib R. Stealing the revolution: violence and predation in Libya. NOREF Report. https://www.clingendael.org/sites/default/files/pdfs/Tabib_Clingendael_NOREF_Stealing%20the%20revoulution_Violence%20and%20predation%20in%20Libya_October%202014.pdf.

[62] Abul-Hajj S. Libyan women: liberated but not yet free. http://www.equaltimes.org/libyan-women-liberated-but-not-yet-free?lang=en#.Vu6MsfmLTIU.

[63] Non-communicable disease risk Factor Collaboration (NCD-RisC).: "World Map - Obesity > BMI > Data Visualisations > NCD-RisC. http://ncdrisc.org/obesity-prevalence-map.html.

[64] Gopalan J. BMI Boom and Economic Collapse in Nauru | Harvard Political Review. https://harvardpolitics.com/world/obesity-in-nauru/.

[65] Alnohair S. (2014). Obesity in Gulf countries. *International Journal of Health Sciences*.

[66] Benjamin K., Donnelly T. (2013). Barriers and facilitators influencing the physical activity of Arabic adults: a literature review. *Avicenna*.

[67] Kanter R., Caballero B. (2012). Global gender disparities in obesity: a review. *Advances in Nutrition*.

[68] Benjamin K., Donnelly T. (2013). Barriers and facilitators influencing the physical activity of Arabic adults: a literature review. *Avicenna*.

[69] Chamieh M. C., Moore H. J., Summerbell C., Tamim H., Sibai A. M., Hwalla N. (2014). Diet, physical activity and socio-economic disparities of obesity in Lebanese adults: findings from a national study. *BMC Public Health*.

[70] Pei L., Cheng Y., Kang Y., Yuan S., Yan H. (2015). Association of obesity with socioeconomic status among adults of ages 18 to 80 years in rural Northwest China. *BMC Public Health*.

[71] Chung S., Popkin B. M., Domino M. E., Stearns S. C. (2007). Effect of retirement on eating out and weight change: an analysis of gender differences∗. *Obesity*.

[72] St-Onge M.-P., Gallagher D. (2010). Body composition changes with aging: the cause or the result of alterations in metabolic rate and macronutrient oxidation?. *Nutrition Journal *.

[73] Morita Y., Iwamoto I., Mizuma N. (2006). Precedence of the shift of body-fat distribution over the change in body composition after menopause. *Journal of Obstetrics and Gynaecology Research*.

[74] High National Election Commission (HNEC) Libya health emergencies and humanitarian update. https://libya360.files.wordpress.com/2012/01/draftelectorallawunofficialtranslation.pdf.

[75] Mozumdar A., Roy S. K. (2004). Method for estimating body weight in persons with lower-limb amputation and its implication for their nutritional assessment. *American Journal of Clinical Nutrition*.

